# A systematic study on the use of multifunctional nanodiamonds for neuritogenesis and super-resolution imaging

**DOI:** 10.1186/s40824-023-00384-9

**Published:** 2023-04-27

**Authors:** Jaeheung Kim, Moon Sung Kang, Seung Won Jun, Hyo Jung Jo, Dong-Wook Han, Chang-Seok Kim

**Affiliations:** 1 Department of Cogno-Mechatronics Engineering, Pusan National University, Busan, 46241 Republic of Korea; 2 Agency for Defense Development, Ground Technology Research Institute, Daejeon, 34186 Republic of Korea; 3 Bio-IT Fusion Technology Research Institute, Pusan National University, Busan, 46241 Republic of Korea; 4 Engineering Research Center for Color-Modulated Extra-Sensory Perception Technology, Pusan National University, Busan, 46241 Republic of Korea

**Keywords:** Nanodiamonds, Neuritogenesis, Super-Resolution Imaging, Photoblinking Direct stochastic optical reconstruction microscopy

## Abstract

**Background:**

Regeneration of defective neurons in central nervous system is a highlighted issue for neurodegenerative disease treatment. Various tissue engineering approaches have focused on neuritogenesis to achieve the regeneration of damaged neuronal cells because damaged neurons often fail to achieve spontaneous restoration of neonatal neurites. Meanwhile, owing to the demand for a better diagnosis, studies of super-resolution imaging techniques in fluorescence microscopy have triggered the technological development to surpass the classical resolution dictated by the optical diffraction limit for precise observations of neuronal behaviors. Herein, the multifunctional nanodiamonds (NDs) as neuritogenesis promoters and super-resolution imaging probes were studied.

**Methods:**

To investigate the neuritogenesis-inducing capability of NDs, ND-containing growing medium and differentiation medium were added to the HT-22 hippocampal neuronal cells and incubated for 10 d. In vitro and ex vivo images were visualized through custom-built two-photon microscopy using NDs as imaging probes and the direct stochastic optical reconstruction microscopy (dSTORM) process was performed for the super-resolution reconstruction owing to the photoblinking properties of NDs. Moreover, ex vivo imaging of the mouse brain was performed 24 h after the intravenous injection of NDs.

**Results:**

NDs were endocytosed by the cells and promoted spontaneous neuritogenesis without any differentiation factors, where NDs exhibited no significant toxicity with their outstanding biocompatibility. The images of ND-endocytosed cells were reconstructed into super-resolution images through dSTORM, thereby addressing the problem of image distortion due to nano-sized particles, including size expansion and the challenge in distinguishing the nearby located particles. Furthermore, the ex vivo images of NDs in mouse brain confirmed that NDs could penetrate the blood–brain barrier (BBB) and retain their photoblinking property for dSTORM application.

**Conclusions:**

It was demonstrated that the NDs are capable of dSTORM super-resolution imaging, neuritogenic facilitation, and BBB penetration, suggesting their remarkable potential in biological applications.

**Graphical Abstract:**

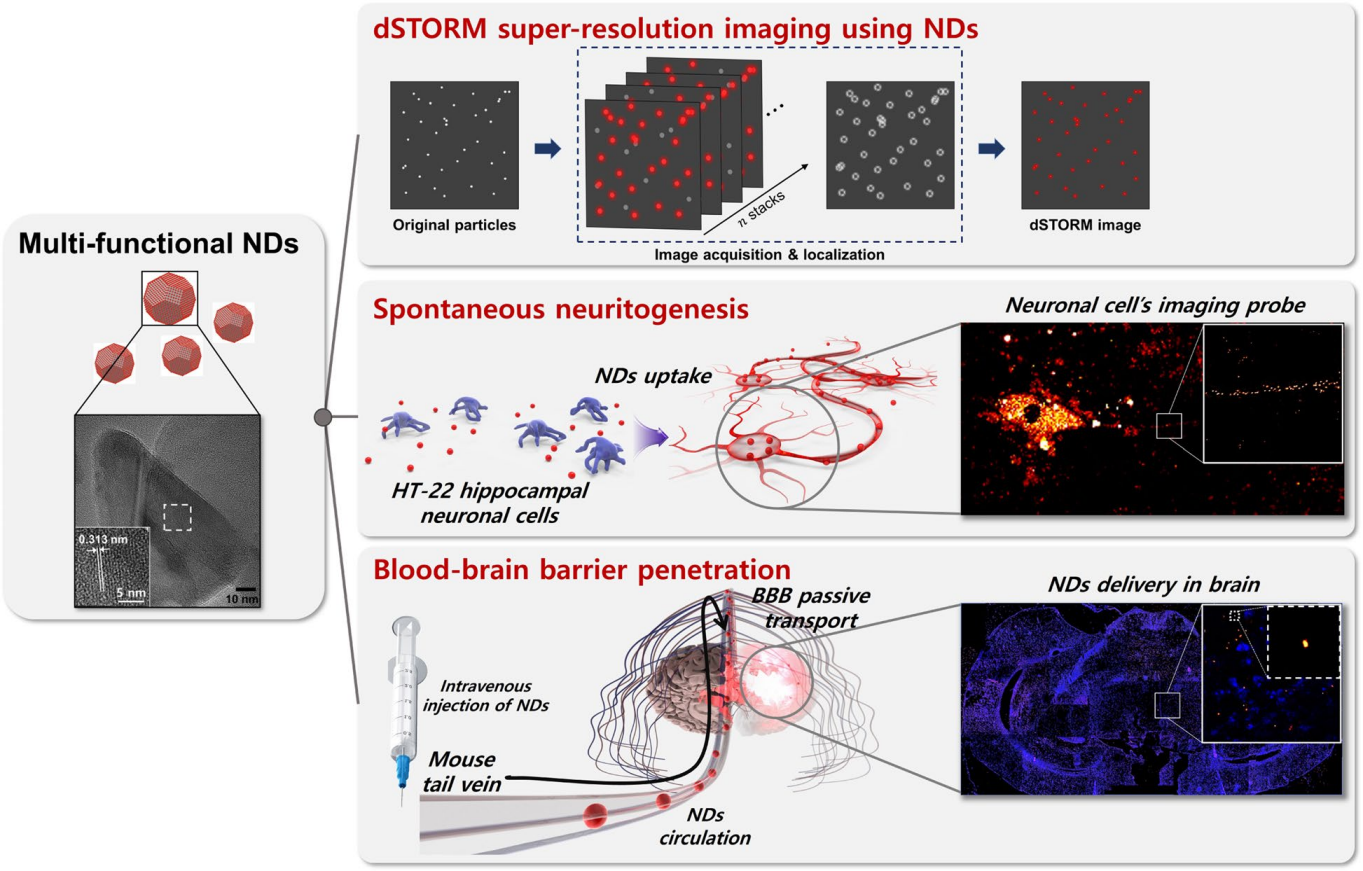

**Supplementary Information:**

The online version contains supplementary material available at 10.1186/s40824-023-00384-9.

## Background

The central nervous system (CNS), which is composed of the brain and spinal cord, is a complex and sophisticated system that regulates the motor and sensory functions of the body. However, the CNS can suffer damage due to internal and external causes (such as stroke, physical damage, infection, tumor, and metabolic and degenerative diseases), leading to structural defects and functional disorders in nerve tissues [1–3]. In particular, as the life expectancy of humans increases, the incidence of neurodegenerative diseases such as Alzheimer's disease, Lewy body dementia, frontotemporal degeneration, Parkinson's disease, and amyotrophic lateral sclerosis increases causing severe social and economic costs [4–7]. The incidence increases exponentially as aging progresses, resulting in severe social and economic costs. Because it is difficult for damaged neurons to spontaneously restore to neonatal neurites, they often fail to form synapses with existing cells for signal network recovery [8, 9]. Among neuronal cells, hippocampal neuronal cells are known to express essential cholinergic markers such as affinity choline transporter, choline acetyltransferase, vesicular acetylcholine transporter, and muscarinic acetylcholine receptors, which are known to be involved mainly in cognitive deficits in neuronal diseases [10]. Therefore, tissue engineering approaches have focused on neuritogenesis to achieve the regeneration of damaged hippocampal cells [11]. In this process, the facilitation of neuritogenesis with precise observation of neurite outgrowth is significant for elucidating the deeper mechanism of neurogenic differentiation.

The need for high-resolution cell observations has triggered technological progress in the field of optical microscopies. However, based on Abbe’s law, the physical limitation of the numerical aperture (NA) of the objective lens poses a limit to the achievable resolution in all optical microscopies [12]. Moreover, owing to the point spread function (PSF) of microscopy, also known as the Airy diffraction pattern, particles are imaged to be larger than their actual sizes. This distortion becomes a serious problem when the PSF overlap is present, which is critical for nanoscale particles. However, in neuronal imaging, it is critical to overcome these limitations to precisely observe nanoscale cellular morphologies [13, 14]. The advances in super-resolution imaging techniques have enabled us to overcome the specific resolution constraints set by optical diffraction limits. Stochastic optical reconstruction microscopy (STORM) is a representative super-resolution fluorescence imaging approach, included in single-molecule localization microscopy family [15, 16]. STORM randomly activates only a subset of reporter fluorophores by stimulating a nearby activator dye to induce each reporter fluorophore into the ‘on’ state in a certain frame; the fluorophores revert to the ‘off’ state when there is no stimulation. This repeated on–off process is referred to as photoblinking. Next, by accumulating a series of frames, each fluorophore is localized and reconstructed with high precision, leading to a super-resolved image. Moreover, several organic fluorophores are reported to exhibit photoblinking on their own, without chemical or systematic modulations, thereby enabling super-resolution imaging called direct STORM (dSTORM) [17, 18].

Novel fluorogenic probes have been actively studied to achieve enhanced fluorescence microscopy [19–23]. In particular, carbon nanoparticles (CNPs) have been investigated as cell imaging probes because of their superior biological and physicochemical properties when compared with those of conventional dyes [24–27]. Furthermore, CNPs have exceptional biological prospects in controlling cellular behaviors, including adhesion, migration, proliferation, differentiation of cells into specific lineages, and maturation of progenitor cells [28–32]. CNPs can exceptionally penetrate the blood–brain barrier (BBB), which has microscale pores to allow only nano-sized materials into the brain [33]. Fluorescent dyes are considered inadequate for tracking in vivo neuronal behaviors owing to their inherent toxicity and low stability. In contrast, CNP-based imaging probes are considered safe and stable. To select the appropriate type of CNPs, it is important to consider both their biological and optical properties that can simultaneously facilitate cellular behavior and enable long-term cell tracking with low photobleaching [34–37].

Among the CNPs, nanodiamonds (NDs) are widely used as fluorescent probes for long-term cell tracking because of their biocompatibility and photostability. The fluorescence of NDs, mostly derived from nitrogen-vacancy (NV) centers, has the unique characteristics of far-red emission and non-photobleaching [38, 39]. Moreover, NV centers, an internal defect in NDs, can be utilized in super-resolution imaging probes owing to their photochromic property, which is caused by the electron transition between NV^−^ and NV^0^ when illuminated by a laser. This photoionization is hypothetically related to photoblinking, which is stochastically converted to a fluorescent (‘on’) or non-fluorescent (‘off’) state during the detection of certain spectra [40, 41]. Because of this phenomenon, NDs can be applied to the dSTORM technique without any additional manipulations, on neither the particle itself nor systemically. Furthermore, recent studies have suggested a neurogenic possibility of ND because it has exceptional cytocompatibility derived from relatively low toxicity, non-generation of intracellular reactive oxygen species (ROS), and high endocytosis efficiency while maintaining plasma membrane integrity [42–45]. Autofluorescence is another hurdle in cell imaging using CNPs. Background autofluorescence, induced by natural emissions from molecules that increase cellular metabolism, often makes it challenging to differentiate between the background and fluorescence signals. The excitation and emission spectra of ND (560 nm /700 nm) detour the autofluorescence range of cells (450–670 nm); hence, it is possible to enhance the image contrast by separating the background signal [46]. While superior cytocompatibility and photostability make NDs promising bioimaging probes, NDs hypothetically possess neuritogenic potential comparable with those of other CNPs.

Herein, we explored the versatile properties of ND for in vitro super-resolution imaging of HT-22 hippocampal neuronal cells with the facilitation of spontaneous neuritogenesis and ex vivo imaging of the mouse brain. The physicochemical and optical properties of the ND were characterized; and we performed the dSTORM process with the photoblinking ND for super-resolution reconstructions of acquired images using two-photon microscopy (TPM) and evaluated the image enhancement. In addition, the use of NDs enabled spontaneous neuritogenesis in HT-22 hippocampal neuronal cells without differentiation factors and induced clear visualization of protruded neurites. Moreover, intravenously injected NDs penetrated the BBBs and reached the entire brain, suggesting their further in vivo brain applications.

## Methods

### Preparation and physico-optical characterization of NDs

Red fluorescent NDs (brFND-100) with more than 1000 NV centers of both NV^−^ and NV^0^ per particle were purchased from FND Biotech (Taipei, Taiwan). These particles offer the advantages of photoblinking and higher fluorescence intensities [38, 41]. Before each experimental run, the ND powders were suspended in deionized (DI) water at 2 mg/mL, sonicated for 1 h, and vortexed for 10 min. The morphology of the NDs was characterized using a 200 kV field-emission transmission electron microscope (FE-TEM, TALOS F200X, Thermo Fisher Scientific, Waltham, MA) on a 200 mesh 125 µm copper grid (Sigma-Aldrich, St. Louis, MO). The selected-area electron diffraction (SAED) patterns and fast Fourier transform (FFT) images of the NDs were processed using Thermo Scientific Velox™. The optical properties of the NDs were investigated using a custom-built TPM. The photoblinking phenomenon of the NDs in every imaging round was indicated by tracking the fluorescence emission of a single particle over time. The wavelength of the laser for two-photon absorption was optimized by measuring each of the two-photon emission intensities at wavelength illuminations of 700–1000 nm but with identical optical powers.

### Custom-built TPM imaging system

**Fig. 1:**
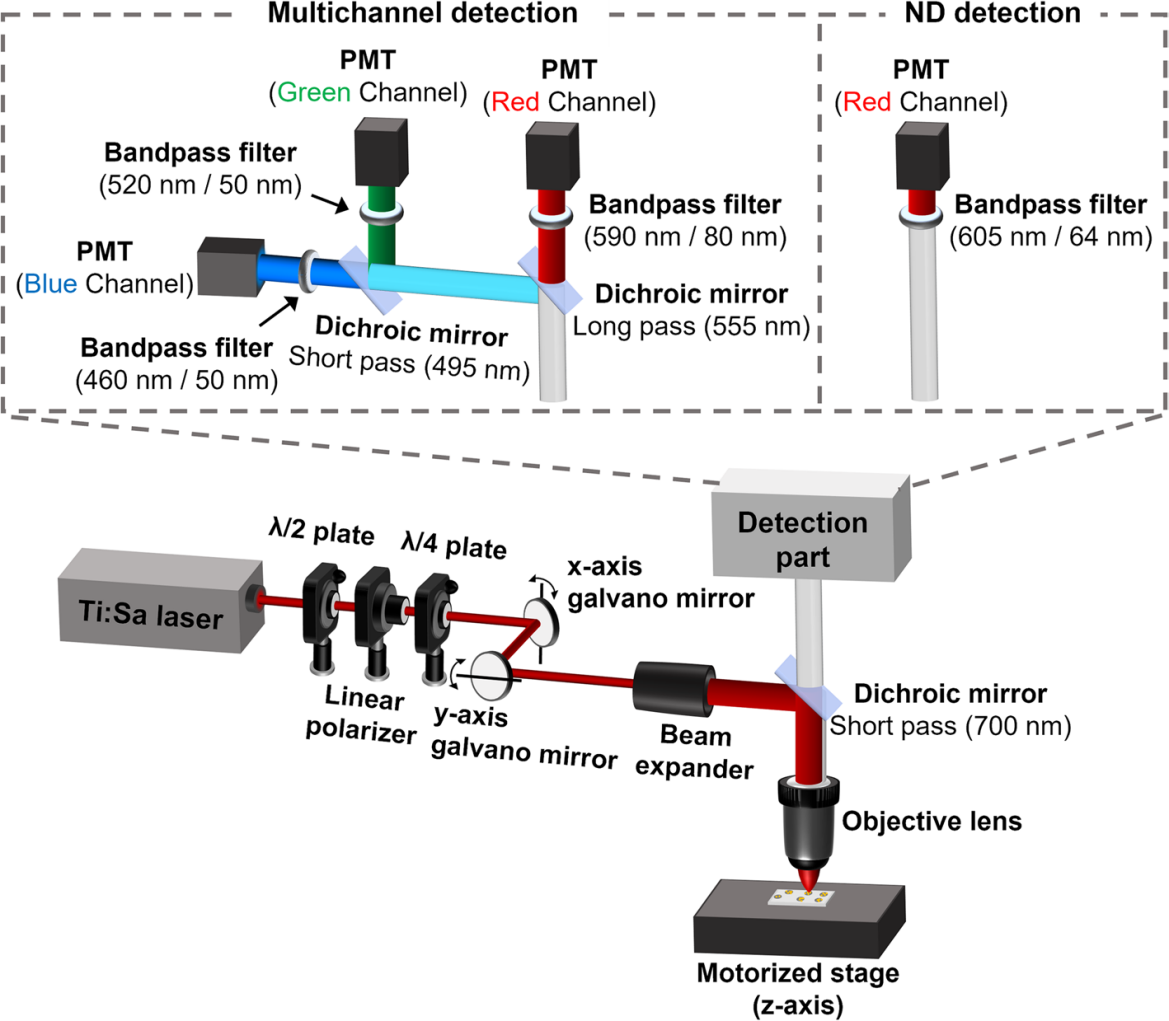
Schematic of custom-built TPM imaging system

The custom-built TPM system used as the main imaging modality and utilized in dSTORM process is depicted in Fig. 1. A Ti:sapphire laser (Chameleon Ultra II, Coherent, Santa Clara, CA) was used in the TPM imaging system, which could modulate wavelengths in the range 680–1080 nm at 1 nm intervals. Because the laser emitted a collimated beam at a specific power, a half-wave plate (AHWP05M-980, Thorlabs, Newton, NJ) and linear polarizer (LPNIR100-MP2, Thorlabs, Newton, NJ) were added to adjust the power. A quarter-wave plate (AQWP05M-980, Thorlabs, Newton, NJ) was additionally located after the linear polarizer to convert the linearly polarized light into circular polarization. Lateral scanning of the focused beam in the samples was performed using a galvanometer scanner (GVS002, Thorlabs, Newton, NJ). The beam expander was composed of a tube lens and a scanning lens, forming a telecentric 4f system to control two fundamental parameters: (a) the expansion of the collimated beam size entering the objective lens, aimed at maximizing the efficiency of the NA of the objective, and (b) the scan angle of the beam into the objective, which determined the size of the field of view (FOV). The 4f system also facilitated stable maintenance of the axial focal plane during lateral scan angle variation [47, 48]. The objective lens determined the size of the beam focus, and the objective with a magnification of 20 × (XLUMPLFLN20XW, Olympus, Tokyo, Japan) was selected because it was water immersion type; thus, it could detect the samples in Dulbecco’s phosphate-buffered saline (DPBS) (Thermo Fisher Scientific, Waltham, MA). In the detection section, three photomultiplier tubes (PMTs) (H10682, Hamamatsu Photonics, Shizuoka-ken, Japan) were used for multichannel detection in the wavelength ranges 434–485 nm (blue), 495–545 nm (green), and 550–630 nm (red). Two dichroic mirrors were placed in front of the PMTs to split the beam based on its respective wavelength bands. Immediately before entering the PMTs, the beams were passed through additional bandpass filters to maintain the wavelength range. Occasionally, a single PMT with a bandpass filter with transmission range 573–637 nm was used when the NDs were used as the only probe for imaging because the wavelength range was determined to be optimum for capturing photoblinking emissions [41].

### Image analysis and reconstruction process for super-resolution image

We used Fiji software, an open-source image processing package based on the ImageJ, for image analysis and processing. The ND-endocytosed cells were reconstructed for super-resolution images using the dSTORM process. This method is based on the self-photoblinking properties of ND. We acquired 100–150 frames of TPM images in the same FOV, corresponding to 220–330 s of acquisition. Each frame captured the stochastic photoblinking of the respective ND particle. Finally, reconstruction was performed using the ThunderSTORM software [49].

### Cell culture and conditions

HT-22 mouse hippocampal neuronal cells were cultured in a growth medium (GM) comprising Dulbecco’s modified Eagle’s medium (DMEM, Welgene, Daegu, Korea) with high glucose containing 10% fetal bovine serum (FBS, Welgene, Daegu, Korea) and 1% antibiotic–antimycotic solution (ABS, 10,000 units of penicillin, 25 μg/mL of amphotericin B, and 10 mg of streptomycin, Sigma-Aldrich, St. Louis, MO) in a 37 °C humidified incubator with 5% CO_2_ atmosphere. The cells were routinely sub-cultured at 70% confluence using a trypsin–EDTA solution (Sigma-Aldrich, St. Louis, MO). Cells from passages 3 to 4 were seeded at an optimized density in each culture plate. To induce neuritogenesis, HT-22 cells were seeded on 24 well plates at 10^4^ cells/well for fluorescence microscope imaging and 6 well plate at 5 × 10^4^ cells/dish for TPM. For positive control with a differentiation medium (DM), a neurobasal medium (NBM, Sigma-Aldrich, St. Louis, MO) containing 2% B-27 supplement (Sigma-Aldrich, St. Louis, MO), 1% L-glutamine (Sigma-Aldrich, St. Louis, MO), 1% N2 supplement, 57 ng/mL epidermal growth factor (EGF, Peprotech, Rocky Hill, NJ), 50 ng/mL basic fibroblast growth factor (bFGF, Peprotech, Waltham, MA), and 1% Abs was added and the dishes were incubated for 10 days in vitro (DIV) [29, 50–52]. To investigate the neuritogenesis-inducing capability of NDs, 250 µg/mL of ND-containing GM and DM were added to the cells and incubated for 10 DIV.

### In vitro cytotoxicity test

The dose-dependent cytotoxicity of NDs was evaluated using a cell counting Kit-8 assay (CCK-8) (Dojindo, Kumamoto, Japan). HT-22 hippocampal neuronal cells were seeded in 48 well plates at a density of 1.5 × 10^4^ cells/well and incubated with various concentrations of NDs for 24 and 48 h. At a predetermined time, the cells were washed three times with DPBS. Subsequently, a ten times diluted CCK-8 assay solution was added to the culture medium, which was incubated for 2 h at 37 °C in the dark. After incubation, supernatants were collected and transferred to new 96 well plates. Absorbance was measured at 450 nm using a SpectraMax 340 ELISA Reader (Molecular Device Co., Sunnyvale, CA). To assess membrane integrity, LDH assay was employed to measure the quantity of lactate dehydrogenase (LDH) that leaked out of the cells. The procedure involved incubating the cells with increasing concentrations (0 to 500 µg/ml) of NDs for 24 and 48 h, and then transferring the supernatant from the treated cells to a new 96 well plate. After adding LDH solution to each well, the plate was incubated for 30 min at 25 °C in the dark, followed by measuring the absorbance at 490 nm using an ELISA Reader.

Cellular oxidative stress induced by NDs was quantified by measuring intracellular ROS generation and measured using a CM-H_2_DCFDA (dichlorofluorescein diacetate) molecular probe (Thermo Fisher Scientific, Waltham, MA), which is an intracellular ROS probe, and a free radical sensor, which is a typical oxidative stress indicator for directly evaluating cellular redox states [53, 54]. HT-22 cells were seeded in 48 well plates at a density of 1.5 × 10^4^ cells/well. NDs were treated for various concentrations and incubated for 24 h. Subsequently, DCFDA 5 µM solution was added, and the cells were incubated for 30 min at 37 °C. Intracellular fluorescence micrographs were captured using a fluorescence microscope (IX 81, Olympus, Tokyo, Japan), and the degree of fluorescence was quantified using Fiji.

### Immunofluorescence staining

The nuclei, F-actins, and neurofilaments of HT-22 hippocampal neuronal cells were visualized through immunofluorescence staining. The neurofilament heavy polypeptide which maintains the neuronal caliber structures was used in the maintenance of neuronal caliber and has been extensively used as a typical marker for neuronal differentiation and neuritogenesis [55, 56]. After 10 DIV, the cells were fixed with a 3.7% formaldehyde solution (Sigma-Aldrich, St. Louis, MO) for 10 min and treated with 0.1% Triton X-100 (Sigma-Aldrich, St. Louis, MO) for 5 min, followed by blocking with a 2% bovine serum albumin (BSA, GenDEPOT, Barker, TX) solution for 30 min. Neurofilaments were immunostained with an anti-neurofilament heavy polypeptide antibody (Abcam, Cambridge, MA). After overnight reaction at 4 °C, secondary goat anti-rabbit IgG heavy and light chains (Abcam) conjugated with fluorescein isothiocyanate (FITC) were reacted for 1 h. Subsequently, the nuclei and F-actins were counterstained with 4,6-diamidino-2-phenylindole (DAPI) (Sigma-Aldrich, St. Louis, MO) 1 μM, and tetramethylrhodamine isothiocyanate (TRITC)-labeled phalloidin 165 nM (Molecular Probes, Eugene, OR). Fluorescence images were captured using a fluorescence microscope (IX 81, Olympus, Tokyo, Japan) and a custom-built t. The degree of fluorescence was quantified using Fiji.

### Animal study and ex vivo imaging

All procedures in the animal experiment are performed according to the National Institutes of Health Guide for the Care and Use of Laboratory Animals and the protocols approved by the Pusan National University–Institutional Animal Care and Use Committee (PNU-IACUC; approval no. PNU-2022–3174). The 8-week-old BALB/c nude mice were bred in pathogen-free facilities. Prior to the experiments, the mice were routinely housed in cages at 20–24 °C. All experimental procedures were examined and approved by the Animal Research Ethics Committee of Pusan National University. The ND solution was intravenously injected through the tail vein at a dose of 0.5 mL/kg body weight at 1 mg/mL dissolved in DPBS, of which dose basis was designed by previous studies [57, 58]. The mice were sacrificed 1 and 10 d after the injection. For histological analysis, the extracted main organs including brain, heart, spleen, kidney, liver, and lung were stained with a Dako cover stainer (Agilent, Santa Clara, CA). First, the organs were soaked in hematoxylin solution (Abcam, Cambridge, England) for 6 h at 60–70 °C and rinsed with DI water three times. Acetic acid (10%) and ethanol (85%) was used to differentiate brains after 2 and 10 h. The brains were soaked thrice in 0.3% ammonia water for bluing. Next, the brains were soaked in an eosin solution for 48 h. Subsequently, the brains were dehydrated using 95% ethanol for 30 min and soaked in xylene for 1 h at 60–70 °C, followed by paraffin blocking for 12 h. Finally, the hematoxylin and eosin (H&E)-stained brains were cut into 15-µm slices, dewaxed in a 60 °C water bath, and mounted with Canada balsam mounting solution (Sigma-Aldrich, St. Louis, MO). For ex vivo immunofluorescence staining, dehydrated 15 µm brain slices were counterstained with 1-μM DAPI for 30 min and rinsed three times with DI water. Images were captured using fluorescence microscope (IX 81, Olympus, Tokyo, Japan) for large FOVs, and custom-built TPM for narrow FOVs.

### Statistical analysis

All variables were tested in three independent experiments, each performed in duplicate using different cultures (*n* = 6). Data are presented as mean ± standard deviation. Before statistical analysis, the data were analyzed for equality of variances using Levene’s test. Multiple statistical comparisons were performed using the Bonferroni test after a preliminary one-way analysis of variance. Asterisks (*, **, *** and ****) indicate statistical significance (*p* < 0.05, 0.01, 0.001, and 0.0001, respectively).

## Results

### Physico-optical characteristics of NDs

**Fig. 2:**
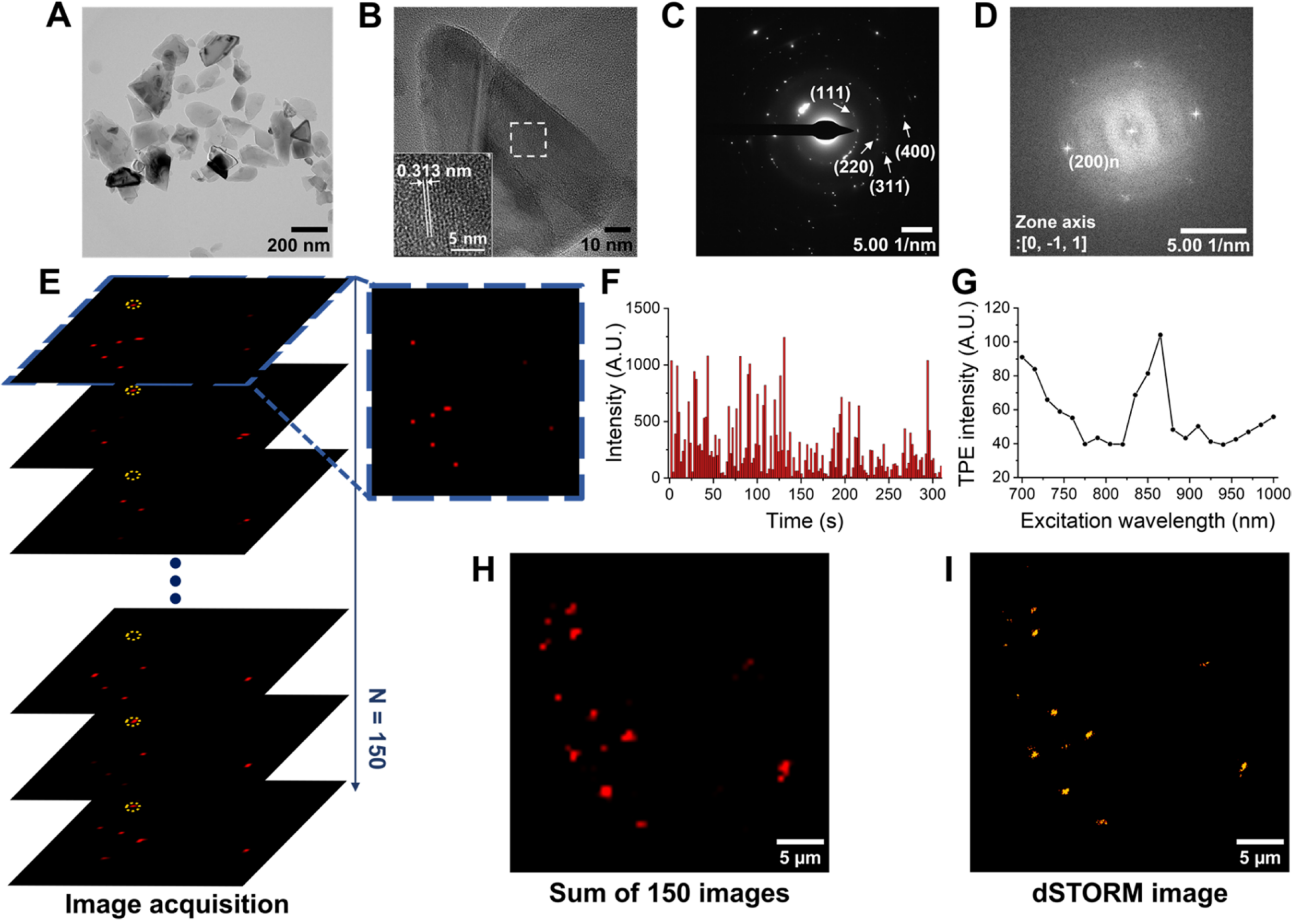
Physico-optical characterization of ND. **A** Morphological characterization of NDs using FE-TEM. **B** Highly magnified FT-TEM image. **C** SAED pattern and **D** FFT image of single ND. **E** TPM image acquisition of ND particles of total 150 frames. Image surrounded by blue dashes depicts the first frame. **F** Fluorescence intensity variation of single ND marked in yellow dotted area in **E** during image acquisition over time. **G** NDs’ two-photon emission intensities depending on the excitation wavelengths. **H** Sum of the signal with maximum intensity in each pixel among 150 frames of **E**. **I** dSTORM image through 150 frames of **E**

Figures 2A–D depict the FE-TEM images of the prepared NDs for morphological characterization. Figure 2A indicates that the NDs were 50–250 nm in diameter and had a uniform general morphology. Figure 2B depicts a magnified image of the ND, indicating that the prepared NDs were not aggregated and maintained their pristine morphologies. The inset in Fig. 2B depicts a detailed analysis, indicating that the white squared lattice spacing was 0.313 nm, which corresponded to the (200) lattice planes of diamond with a face-centered cubic structure [59]. It is evident that the SAED pattern of the prepared ND has four strong diffraction rings, representing the (111), (220), (311), and (400) planes of diamond (Fig. 2C) [60]. The FFT image depicts a single-crystalline n-diamond along the (110) zone axis (Fig. 2D). The (200) forbidden plane indicates that the prepared ND has an n-diamond structure (Fig. 2D). Therefore, the FE-TEM and FFT images clearly visualized the (111) and (200) lattice planes, indicating that the prepared NDs were single-crystalline n-diamonds [59].

A total of 150 frames of TPM images were acquired (Fig. 2E), and they were reconstructed to obtain the dSTORM image (Fig. 2I). Only a single PMT with a 605/54 nm bandpass filter was used for this optical characterization because the NDs were found to have clear photoblinking in the wavelength range [41]. We observed the photon-counted fluorescence intensity of the ND over time to verify on–off state cycles by tracking the signals of ND in the area marked in yellow dots in Fig. 2E. During image acquisition over time, the variation in the fluorescence intensity indicates photoblinking of the ND, as depicted in Fig. 2F. The intensity fluctuation indicates the contrast between the ‘on’ and ‘off’ states of the ND. We analyzed the dSTORM image of the NDs acquired using TPM and compared it with the sum of the signal with maximum intensity in each pixel among the total 150 frames (Fig. 2H) because a single frame of the image could not visualize the whole ND particles as depicted in the image surrounded by blue dashes in Fig. 2E owing to the photoblinking. Note that a dot that looked like a single particle in Fig. 2H is identifiable as several particles in the dSTORM image (Fig. 2I). Moreover, to estimate the optimal wavelength of the laser for two-photon emission, the fluorescence intensity was measured using TPM by changing the excitation wavelength in the range 700–1000 nm at the same optical power, as depicted in Fig. 2G. An excitation wavelength of 860 nm was determined to be optimal for the TPM imaging of NDs.

### Cytotoxicity of NDs

**Fig. 3:**
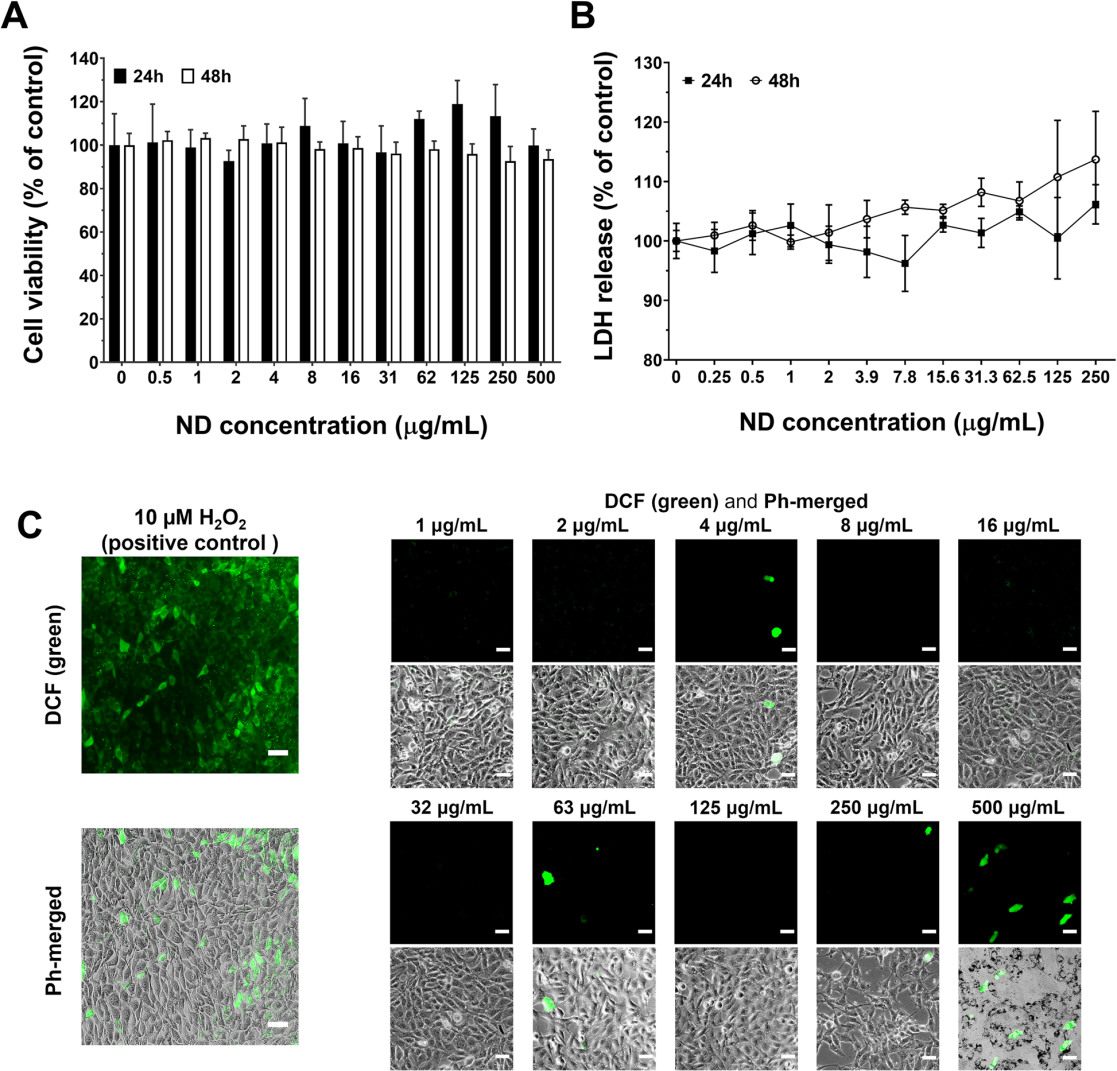
Cytotoxicity of NDs on HT-22 hippocampal neuronal cells. **A** CCK-8 assay for cytotoxicity profiling of HT-22 cells. DCFDA assay for evaluation of intracellular ROS generation induced by ND uptake for **B** LDH release profiling of HT-22 cells. **C** DCFDA assay for evaluation of intracellular ROS generation induces by ND uptake. Scale bars denote 50 µm

The cytotoxic effects of the NDs on HT-22 hippocampal neuronal cells were evaluated to optimize the treatment dose (Fig. 3). The viability of HT-22 cells treated with NDs was examined using the CCK-8, LDH, and DCFDA assays. From the CCK-8 assay, the cell viability exhibited no decreasing tendency up to 500 µg/mL, suggesting that the NDs had no significant dose-dependent cytotoxicity on HT-22 cells up to 500 µg/mL both at 24 and 48 h (Fig. 3A). Figure 3B demonstrates that there was no noticeable release of intracellular LDH up to 500 µg/mL at both 24 and 48 h. Typically, LDH release rises with greater cell membrane damage, but our findings revealed that the LDH release for ND treatment remained consistent within the 0–500 µg/mL range. Therefore, the LDH test affirmed that ND loading did not impair cell membrane integrity. Subsequently, cellular oxidative stress by intracellular ROS generation, one of the most common cytotoxic mechanisms of nanomaterials, was assessed using a general oxidative stress indicator (CM-H_2_DCFDA) (Fig. 3C). When DCFDA enters cells, the two ester bonds in their structure are broken. Subsequently, the produced H_2_DCF is accumulated by intracellular ROS and is represented as highly fluorescent DCF (green fluorescence in Fig. 3C) [61]. When the cells were treated with 10 µM H_2_O_2_, green fluorescence of DCF was observed, indicating spherical cell morphologies. The 500 µg/mL NDs induced a couple of exhibitions of green-fluorescent DCF and altered the pristine spindle-like morphology of HT-22 cells. In contrast, NDs ≤ 250 µg/mL did not induce remarkable green-fluorescent DCF and maintained the pristine morphology of the HT-22 cells. Although 500 µg/mL NDs did not decrease cell viability after 24 h of incubation, the morphological alteration and intracellular ROS by 500 µg/mL NDs could adversely affect the cellular behaviors in long-term culture. Further experiments were performed with 250 µg/mL of NDs.

### Neuritogenic effects of NDs on hippocampal neuronal cells

**Fig. 4:**
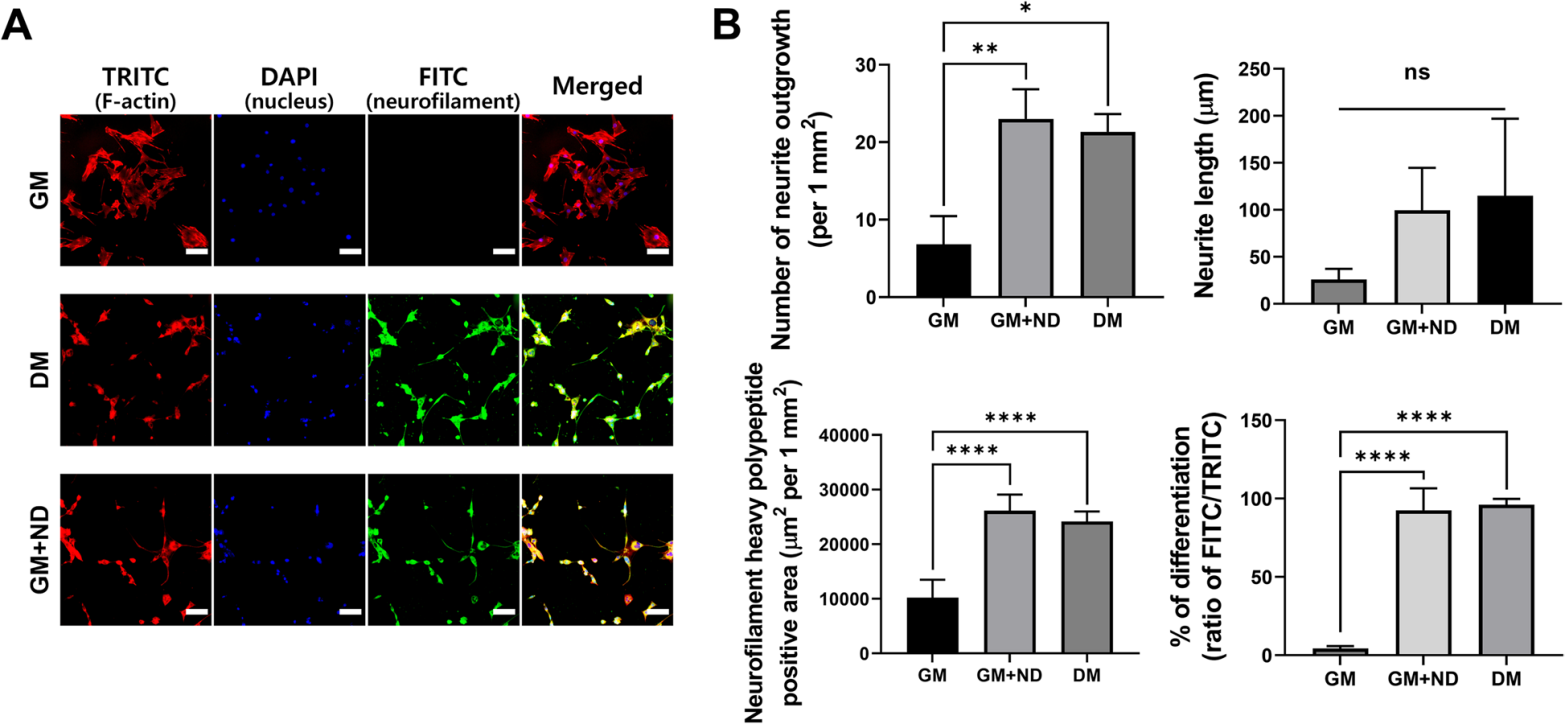
Neuritogenic effect of NDs on HT-22 hippocampal neuronal cells. **A** Fluorescence microscope images of ND-treated HT-22 cells at 10 DIV cultured in GM, DM, and GM + ND (250-µg/mL ND-treated GM). **B** Quantified evaluation of HT-22 cells at each group indicating the number of neurite outgrowths, average neurite length (µm), neurofilament heavy polypeptide positive area (µm^2^/mm^2^), and the percentage of differentiation (ratio of FITC/TRITC). Scale bars denote 100 µm. Asterisks denote statistical significances compared to GM

Immunofluorescence staining was performed to evaluate the neuritogenic effects of NDs (Fig. 4). After 10 d of culture, HT-22 cells cultured in GM, DM, and GM + ND (250 µg/mL ND-treated GM) were imaged. The F-actin (TRITC), nucleus (DAPI), and neurofilament (FITC) were visualized through fluorescence microscope (Fig. 4A). Based on Fig. 4A, the degree of differentiation was measured (Fig. 4B). The number of neurite outgrowths per 1 mm^2^ of GM + ND increased 3.4-fold when compared with that of GM. The average neurite length slightly increased in ND-treated group. Quantitative analysis of FITC denoting the neurofilament heavy polypeptide positive area indicated that DM and GM + ND demonstrated significantly (*p* < 0.001) enhanced signals compared with that of GM. The ratio of the fluorescent area of FITC per TRITC denoted the percentage of differentiation and exhibited similar trends, indicating that DM and GM + ND significantly (*p* < 0.001) promoted neuritogenesis, while GM itself could not. These results suggest that NDs not only facilitate spontaneous neuritogenesis in HT-22 cells but also enhance the length and number of neurite outgrowths.

### Cellular uptake of NDs visualized through TPM

**Fig. 5:**
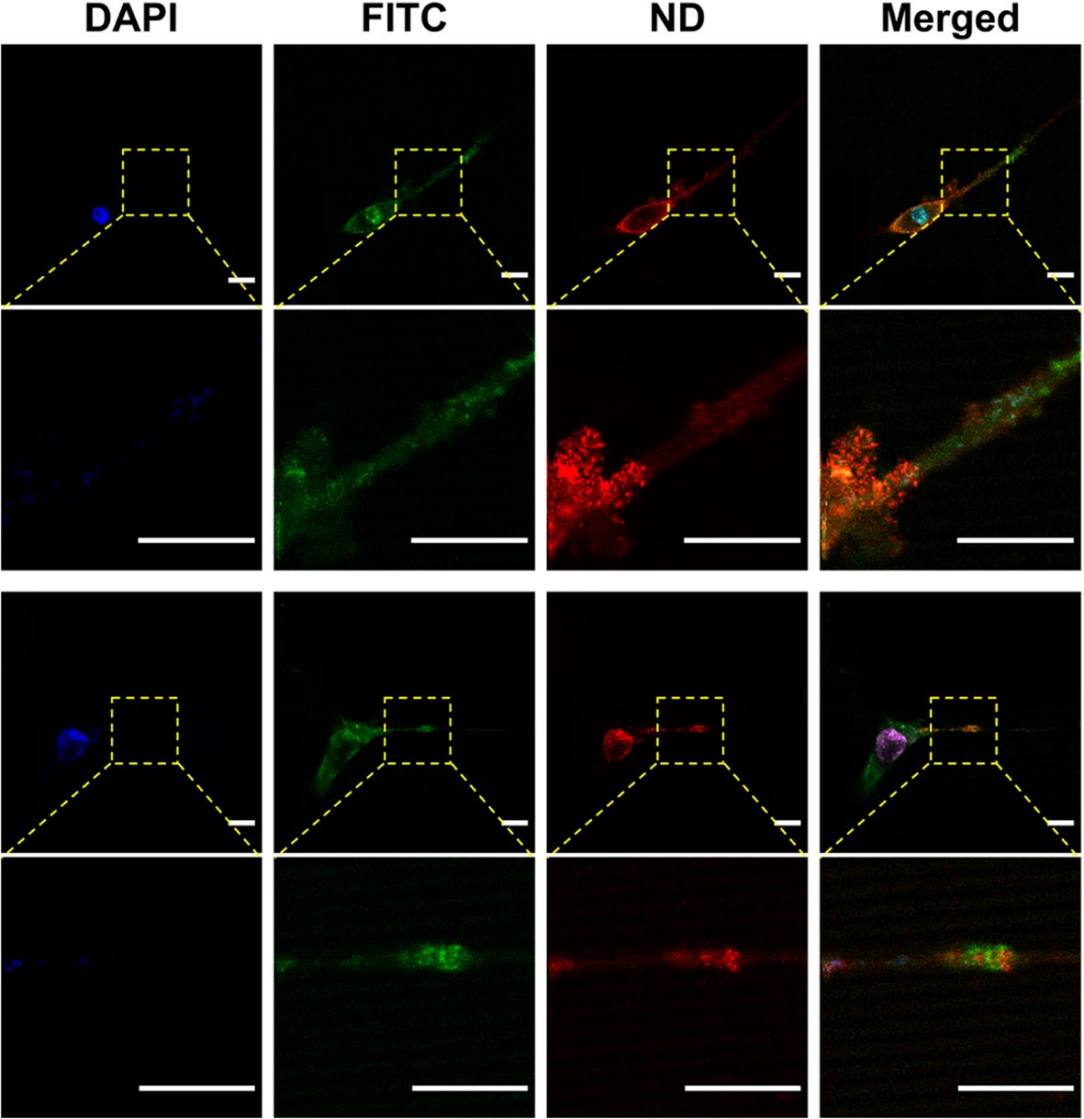
TPM images of immunofluorescence (green and blue channels) and NDs (red channel) endocytosed by HT-22 hippocampal neuronal cells. Images in second and fourth lines exhibit the magnified images of areas marked in yellow dashed boxes of upper images to illustrate neurites in detail. Scale bars denote 20 µm

To evaluate degree of endocytosis of NDs in HT-22 cells, TPM images of immunofluorescence-stained cells were investigated and compared with ND fluorescence signals to evaluate the suitability of NDs as TPM imaging probes (Fig. 5). HT-22 cells were cultured in the ND solution, and the nuclei and neurofilaments were stained with DAPI (blue) and FITC (green), respectively. The lower images represent magnified areas marked with yellow dashed boxes. The lower images in Fig. 5 denote the magnified areas marked as yellow dashed boxes and illustrate neurites in detail. NDs were found to be distributed throughout the cells except for nuclei (DAPI) by comparing the signals in the green (FITC) and red (ND) channels, as visualized in the merged images of Fig. 5. Evidently, the NDs were observed even in newly developed long neurites. We found that the NDs were only visualized in the soma, not in the nucleus, because the nuclear pore complexes passed only through small polar molecules, ions, and macromolecules such as proteins and RNAs. This suggests that NDs are not attached to the membrane but are laden into the cytoplasm through endocytosis.

### In vitro dSTORM super-resolution imaging of hippocampal neuronal cells

**Fig. 6:**
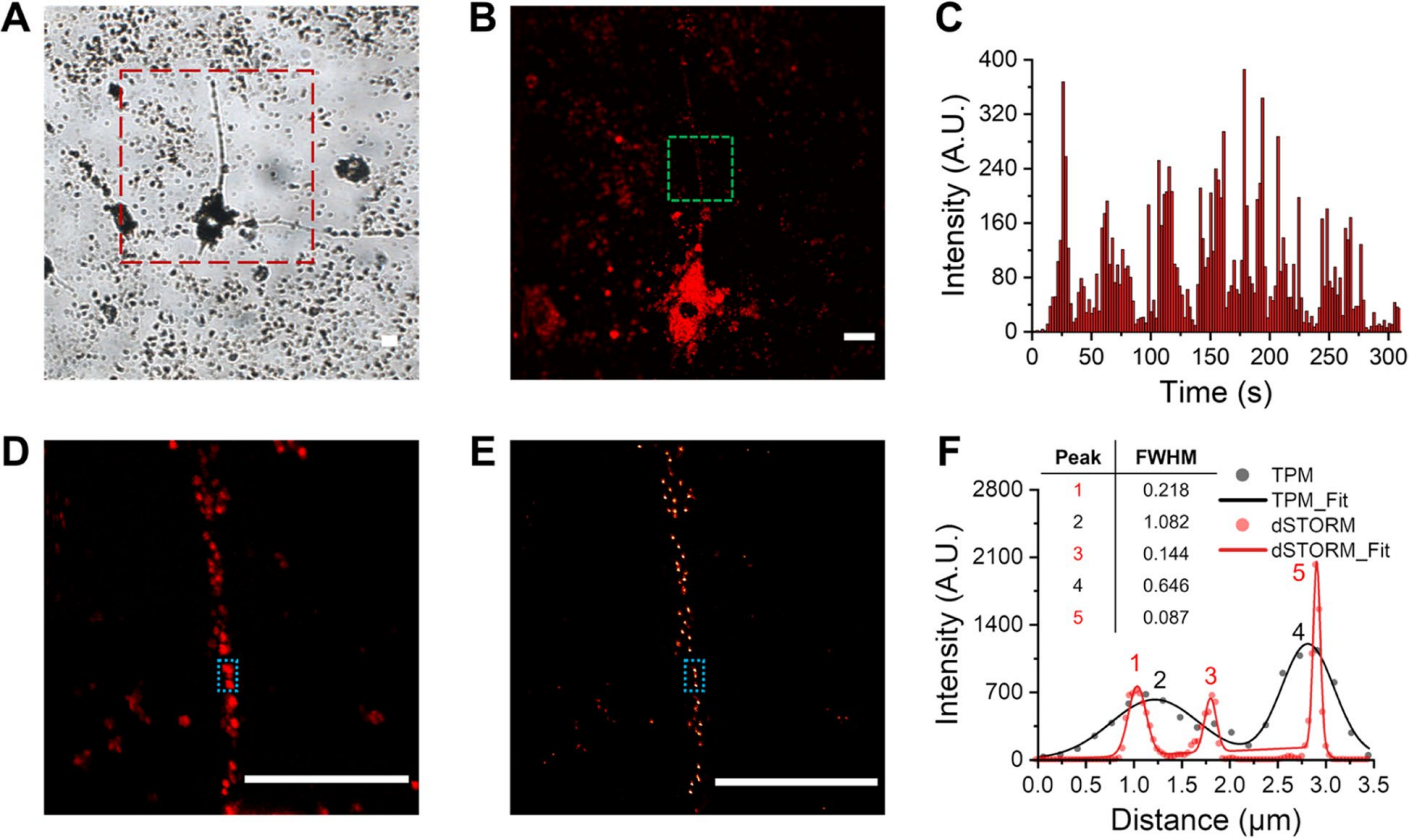
Differentiated single HT-22 hippocampal neuronal cell imaged only with NDs. **A** Optical microscopic bright field image. **B** TPM image of red dashed area in **A**. **C** Intensity variation in one pixel while acquiring 150 frames of green dashed area in **B**. **D** TPM image, indicating the maximum intensity of each pixel among 150 frames of green dashed area. **E** dSTORM image reconstructed through 150 TPM images. **F** 2D cross-sectional intensity profiles marked in blue dotted box. Scale bars denote 20 µm

After verifying that the NDs were well endocytosed in HT-22 cells, images were obtained using only the NDs as fluorescence probes (Fig. 6). During the incubation period, NDs were endocytosed by HT-22 cells and observed to be distributed throughout the whole cell, including the soma and neurites. The TPM image of the ND-laden HT-22 cell (Fig. 6B) visualizes the region marked with the red dashed box in the bright field image (Fig. 6A). For a distinctive comparison of neurite visualizations between the original TPM and dSTORM images, higher magnification images were obtained, as depicted in Fig. 6D, whose FOV is indicated by the green dashed box in Fig. 6B. A total of 150 frames were obtained over time through TPM to perform the dSTORM process. To prevent missing the ‘off’ state portions of the NDs owing to the photoblinking phenomenon, Fig. 6D displays the maximum intensity in each pixel of the 150 frames. To evaluate the photoblinking process in ND-laden neuronal cells, we tracked the intensity variation of a single pixel where the ND existed during 150 frames of image acquisition over time (Fig. 6C). The photoblinking property was retained with a high contrast of ‘on’ and ‘off’ states when attached to neuronal cells, which is suitable for applying to the dSTORM process. Therefore, the dSTORM image, as depicted in Fig. 6E, was reconstructed, and the size expansion and distortion of the NDs due to the PSF and deficient resolution of the TPM were resolved. For further observation, 2D cross-sectional intensity profiles of the blue dashed areas in Figs. 6D–E were analyzed, as depicted in Fig. 6F, where the TPM and dSTORM data are distinguished in black and red, respectively. The dotted plots depict the original intensities of the images, whereas the line plots indicate the Gaussian-fitted forms. While the integrated Gaussian peaks represent the particles with PSFs of the respective images, a blurred and undistinguished particle (peak 2) in the TPM image could be identified as two distinctive particles (peaks 1 and 3) after the reconstruction. Moreover, the full width at half maximum values represent the settlement of the size expansion in the TPM owing to PSF. In particular, the PSF of the identical particle (peak 4 in TPM and peak 5 in dSTORM) was measured to be nearly 7.43 times smaller in dSTORM, suggesting that the dSTORM image can better resolve each particle, whereas the TPM image suffers from precise imaging owing to the insufficient resolution for nano-sized particles.

### Ex vivo mouse brain imaging

**Fig. 7:**
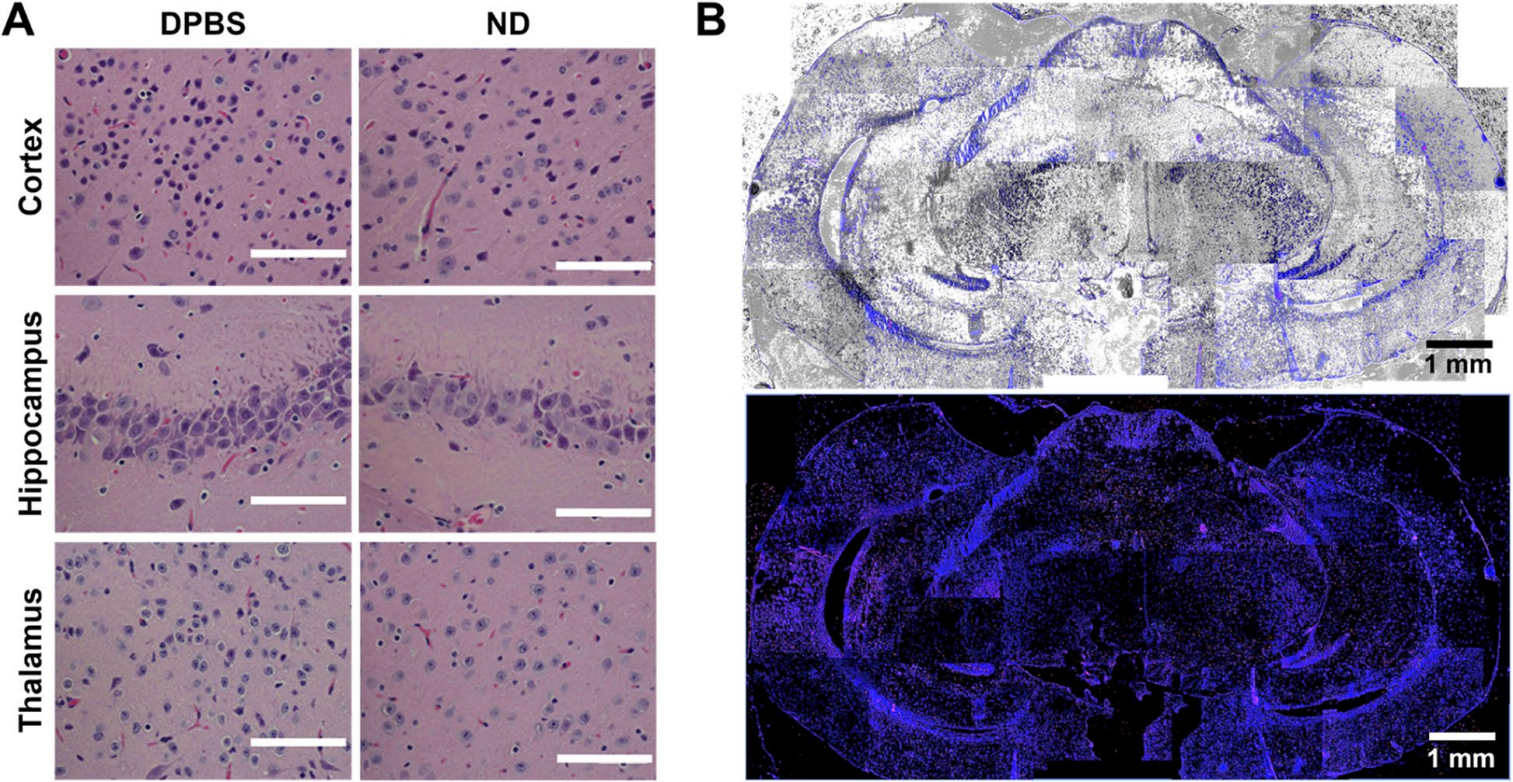
DPBS or ND-injected H&E-stained brain of the mouse after 1 d post-injection. **A** Microscopic examination of horizontal sections of H&E-stained brains including cortex, hippocampus, and thalamus. **B** Immunofluorescence-stained brain images of ND-injected mouse using fluorescence microscope by combining 30 individual 40 × images. Merged version of phase contrast, DAPI, and ND (upper) and merged version of DAPI and ND (lower). Scale bars without value denote 100 µm

To assess the potential of NDs for application to ex vivo brain imaging, the in vivo biosafety of NDs in the cortex, hippocampus, and thalamus was evaluated. Specimens of 8-week healthy BALB/c nude mice were intravenously injected with a single dose of NDs in 20 µL at 1 mg/mL. In both 1 and 10 d post-injection, ND-injected brain did not exhibit noticeable abnormalities/lesions, histomorphological changes, or apoptosis of cells when compared with the 20 µL DBPS-injected mice (Figs. 7A and S1A) [62, 63]. The native structures and cell morphologies of the cortex, hippocampus, and thalamus were not denatured within 10 d post-injection, indicating that the penetrated NDs with long-term safety in brain tissues [64]. Furthermore, we investigated the effects of NDs on main organs after 10 d post-injection as shown in the H&E staining images (Fig. S1B). The main organs (i.e. heart, kidney, liver, lung, and spleen) of ND-injected mice exhibit little abnormalities or lesions which are negligible difference with DPBS-injected groups. The findings provide additional evidence supporting the notion that NDs have good biocompatibility, indicating that they cause minimal acute and chronic toxicity due to their rapid clearance from the body [57]. On the other hand, NDs were passed through the BBB and distributed in the whole area of the brain, indicating that the brain tissues were not damaged by the accumulated NDs (Fig. 7B). The results of the in vivo toxicity analysis in Fig. 7A suggested that the NDs had reasonable biocompatibility during 24 h of injection, suggesting their suitability as ex vivo imaging probes for the brain.

**Fig. 8:**
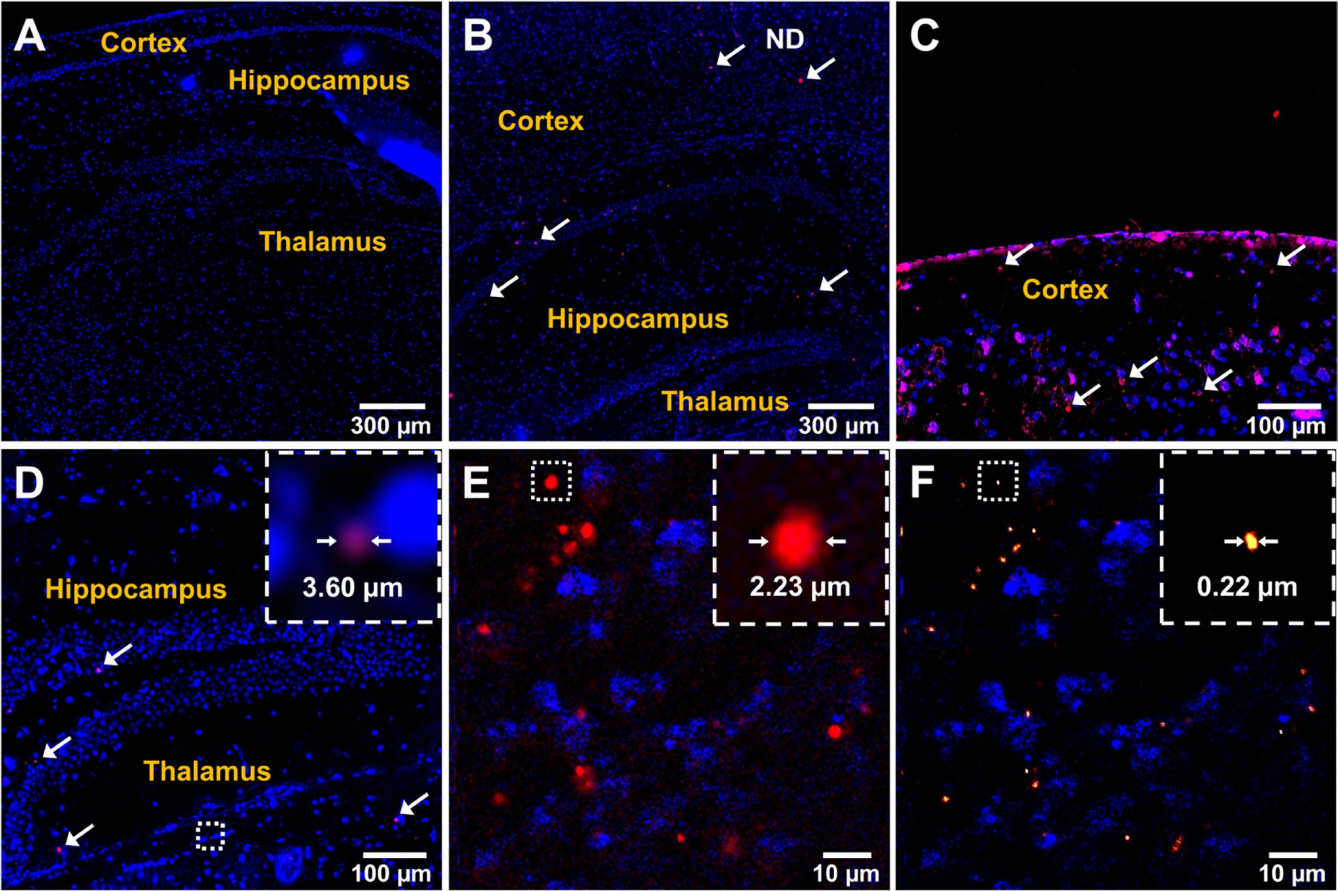
Ex vivo brain imaging after 24 h of ND intravenous injection. Fluorescence microscope images of **A** 20 µL PBS-injected, **B** 20 µg/20 µL ND-injected group, **C** cortex, **D** hippocampus, and thalamus of 20 µg/20 µL ND-injected group. **A**–**D** are visualized using fluorescence microscope. **E** TPM image of the hippocampus of 20 µg/20 µL ND-injected group with high magnification. **F** dSTORM image reconstructed through 150 TPM images. White dotted boxes denote enlarged images of marked areas containing a single ND

Finally, we investigated the feasibility of using the NDs as optical imaging probes for organs. The mouse brain, which has BBBs so that only nano-sized particles can penetrate, was selected to demonstrate the feasibility of NDs as long-term optical imaging probes, penetration of BBBs as a NP, and super-resolution imaging application (Fig. 8). We extracted the brain of the mouse, which had been intravenously injected with NDs for 24 h. The brain was sliced at 15 µm widths for ex vivo fluorescence imaging. For better analysis, the brain slices were stained with DAPI (blue fluorescence) to visualize the cell nuclei. Figures 8A–D represent the fluorescence microscope images of the brain slice with a relatively wide FOV, where the structures of the cortex, hippocampus, and thalamus are recognizable. Figure 8A depicts the 20 µL DBPS-injected control group without the ND injection. The other images in Fig. 8 depict the experimental group with the injection of 20 µg/20 µL ND solution. The NDs successfully penetrated the BBBs and were imaged in the area where nuclei were not located, as depicted in Fig. 5. To assess the improvement in the resolution in various fluorescence imaging techniques, we compared the three images obtained through conventional fluorescence microscope, TPM, and dSTORM based on TPM, as depicted in Figs. 8D–F, respectively. For a clear comparison, cropped images indicating the white dashed boxes in the original images were added, which contained a single ND in the region of interest. TPM achieved a relatively high resolution compared with that of the conventional fluorescence microscope with a reduced blurring phenomenon. However, it still had image distortion owing to the size distortions of the NDs’ actual sizes (≤ 250 nm) caused by the insufficient resolution and PSF. The dSTORM image based on 150 frames of TPM images presented a significant improvement in resolution with settled size distortion. The cropped image of the dSTORM in Fig. 8F indicates that the width of the imaged single ND is measured to be 220 nm, which is close to the actual size and indicates that it remarkably overcame the resolution limit. These measurements confirmed that NDs could penetrate the BBBs and micro-vessels for potential in vivo neuronal applications in the brain and could also be utilized spontaneously for a super-resolution imaging probe.

## Discussion

NDs are a new class of nanomaterials that have emerged as promising imaging agents due to their unique properties compared with other NPs. NDs are made of carbon with the size of 5–100 nm, high surface area, and unique surface chemistry [65]. They are also highly biocompatible, non-toxic, and exhibit superior photostability compared to other fluorescent materials [38, 39]. Additionally, they have the potential for multifunctionality due to their surface functionalization capability [66]. Conventional conjugated polymer NPs are organic fluorescent materials that can emit light upon excitation. They are widely used for bioimaging due to their high brightness and biocompatibility because conjugated polymers have a rigid backbone structure and a large number of delocalized π electrons, which can undergo excited-state intramolecular proton transfer (ESIPT) to emit bright fluorescence and can easily be coated with biocompatible materials, such as polyethylene glycol (PEG) [67, 68]. However, they have limited photostability and can undergo photobleaching under prolonged excitation, which can reduce their imaging quality and accuracy. Aggregation-induced emission (AIE) luminophores are a class of luminophores that exhibit bright fluorescence upon aggregation. They are a promising alternative to conventional fluorescent dyes because of their superior photostability, high brightness, and resistance to photobleaching owing to their aggregation-induce emission behavior [69, 70]. The rigid and planar molecular structures allow AIE luminophores to exhibit strong fluorescence emission upon aggregation, but it also makes them difficult to dissolve in water and other biological media. To improve their solubility and biocompatibility, researchers often modify the surface of AIE luminophores with hydrophilic functional groups, such as PEG or carboxylic acid groups. However, these modifications can increase the size of AIE luminophores, which may limit their ability to penetrate into cells and tissues and reduce their biocompatibility ([71]. Overall, while AIE luminophores have many desirable properties for bioimaging, low biocompatibility may limit their application in some biological systems (Table 1).

**Table 1:** Comparisons of conjugated polymers, AIE luminophores and NDs as fluorescent probes

NP	Size	Photostability	Brightness	Biocompatibility	Potential for Multifunctionality	Ref
NDs	5–100 nm	Superior	High	High	High	[38﻿^39^, ^66^]
Conventional conjugated polymers	10–100 nm	Limited	High	High	Limited	[67, 68]
AIE luminophores	2–100 nm	Superior	High	Low	Limited	[69, 70]

The optical characteristics of ND, which make it suitable for dSTORM applications, were observed through TPM. (d)STORM exploits the photoblinking properties of some fluorophores, which contain a repeated cycle between the ‘on’ and ‘off’ states randomly under certain conditions and allow distinguishing each of their emitted signals. Originally, STORM was performed using an additional activation dye for the stochastic activation of the reporter dye through energy transfer. Owing to the optical property of photoblinking, NDs can be used as super-resolution biomarkers for dSTORM. To the best of our knowledge, (d)STORM is reported to have the highest resolution (< 8 nm) to date among optical imaging systems because the resolution can be pushed to its limit if a sufficient number of images are acquired for precise localization [16, 72, 73]. In single-photon absorption, the NDs were found to emit blinking fluorescence by repeatedly displaying ‘on’ and ‘off’ states over time. The NV center in the ND is a natural paramagnetic impurity composed of a nitrogen atom close to the voids in the lattice. It has three different states: negative charge (NV^−^), neutral charge (NV^0^), and positive charge (NV^+^). Each state exhibits a different emission spectrum. Consequently, the photoblinking mechanism is predominantly thought to be caused by the conversion between the NV^−^ and NV^0^ states owing to photo-induced electron transfer. In this experiment, each ND particle was individually observed at the respective frames until sufficient localization was accumulated to form a reconstructed image. Through this collective process with multiple accumulated images, each particle was reconstructed using a super-resolved localization algorithm with high precision. The photoblinking phenomenon over time in which more ND particles are distributed is shown in the supplementary video S1. The localization precision in (d)STORM imaging is affected by several factors, including the PSF and the signal-to-background ratio of the imaging system [74]. TPM has demonstrated remarkable performance in terms of localization precision and SBR compared to other microscopy techniques [75]. Therefore, dSTORM imaging using TPM is expected to yield better localization precision and resolution. The use of multiphoton excitation through the near-Infrared II (NIR-II) light can expectedly improve the performance of dSTORM. Multiphoton applications in the NIR-II region takes advantage of the low absorption and scattering coefficients in this region, allowing light to penetrate through thick tissue samples [76]. This approach can potentially provide deeper penetration into biological samples while maintaining high localization precision and resolution.

Recent studies have proposed several hypotheses on how NDs can facilitate neuritogenesis and neurogenic differentiation of neuronal cells. Chen et al. demonstrated that surface topography with NDs could facilitate the development of primary neurons in both the central and peripheral nervous systems [77]. The ND-coated surface downregulated the expression of microRNA (miR6236), an inhibitor of neuronal development and regeneration. Taylor et al. also observed neurogenic differentiation of neuronal stem cells on oxygen-terminated ND monolayer [48]. Oxygen-terminated ND monolayer promoted neurogenic differentiation of human neuronal stem cells leading to the increase in the neurite length, degree of branching, and density of neurites expressing MAP2 by its neurogenic effects. It has been suggested that these exceptional characteristics of NDs facilitate extracellular signaling and endow hNSCs with the native stem cell niche to promote neuronal differentiation. ND-treated surfaces have been suggested to enhance surface roughness. F-actin structures on stiff substrates are obstacles to the protrusion of microtubules from the leading edge of the cell body, leading to a large area of F-actin meshworks and protrusion of shorter F-actin bundles, upregulating the neuritogenesis stage [78]. Moreover, because of excellent electrical conductivity of NDs, ND-coated surfaces support the establishment of electrically active neural networks and mature neurons [79]. It has been demonstrated that an ND layer can support initial cell attachment, neuritogenesis, and cell-autonomous neuronal excitability without any protein coating [79]. The expressions of the neurotrophic factor (BDNF), mitochondrial transcription factor-A (TFAM), phosphorylated signal transducer, and activator of transcription-3 (p-STAT3) are known to be upregulated by NDs, thereby facilitating neuritogenesis and maturation of neural cells [80]. Moreover, the expression of inducible nitric oxide synthase (iNOS), nuclear factor-kappa B (NF-κB), and caspase-3 (casp-3) is suppressed, suggesting the neuroprotective effects of NDs on hippocampal neuronal cells [81].

Some previous studies have discussed the neurogenic effects of carbon nanomaterials. Graphene is known to increase cell membrane cholesterol and promote neurotransmission by increasing the number, release probability, and recycling rate of synaptic vesicles [82]. It also promotes axon elongation by promoting hyperpolarization, axonal stretching, and retrograde transport inhibition, without hindering the correct NGF uptake to preserve neuron survival [83]. Nanofiber matrices containing carbon nanotubes were also introduced to enhance PC12 cell proliferation and neuritogenesis by upregulating neuritogenesis-inducing genes, including MAP1b, GAP43, and NF-L [84]. Although there are minor differences, most carbon nanomaterials share analogous biological properties because of the promotion effect of carbon elements on cellular behavior [85–87]. Although the exact mechanisms of NDs in neuritogenesis are still unknown, the similar biological effects of carbon nanomaterials could be applied to hypothesize the neuritogenic effect of NDs.

When compared with STED and STORM, the feasibility of dSTORM for bioimaging has been highlighted in terms of a lower laser power illuminating the samples owing to the uselessness of an additional laser, which also enables clear visualizations of cellular structures, organelles, proteins, and even genes in super-resolution. Recent studies have suggested the superiority of dSTORM applications in bioimaging. Nizamudeen et al. established a dSTORM process to observe stem cell-derived extracellular vesicles in super-resolution using DiD (C_67_H_103_CIN_2_O_3_S) as photoblinking fluorophores. Because STORM techniques require long image acquisition periods, they demonstrated the use of other probes to track the fast-occurring subcellular events during cell imaging [88]. Zhang et al. designed a nucleus-specific probe, namely HoeSR, by conjugating sulforodamine and Hoechst for dSTORM super-resolution imaging of nucleus DNA. Owing to the highly resolved imaging performance of dSTORM, the nuclear nanostructures of the cells were observed in detail [89]. Although dSTORM is hypothetically beneficial for bioimaging among optical super-resolution techniques, it lacks appropriate fluorophores with photoblinking property, biocompatibility, and non-photobleaching. The demonstration of the multifunctional applications of NDs in neuronal imaging as super-resolution imaging probes and neuritogenesis promoters, which could even penetrate the BBB through intravenous injection, connotes its potential for wide applications in in vivo bioimaging and facilitation of neuritogenesis.

## Conclusions

The multifunctional applications of NDs as neuritogenesis promoters and super-resolution imaging probes were proposed in this study. We experimentally found out NDs also activate the neuritogenesis as several types of CNPs, which does not occur without additional differentiation medium during neuronal cell’s incubation period. Since NDs are considered as promising optical biomarkers owing to their optical stability and non-toxicity, the discovery of NDs’ role that influences the neuronal outgrowth development is of great significance compared to other CNPs, which have some restrictions on cell imaging due to their toxicity. At the same time, NDs have a unique feature of emitting photoblinking fluorescence, which facilitated resolving the image distortion from the PSF and distinguished the particles located closer than the optical diffraction limit. In addition, NDs were evaluated as superior neuronal imaging trackers after identifying ND’s good uptake capability in neuronal cells during the cell culture. Optical properties of NDs, including photoblinking, were retained even after being endocytosed in neuronal cells, facilitating the dSTORM process in neuronal applications. Moreover, the intravenously injected NDs that penetrated BBBs remained stable and did not induce severe toxicity in the brain, suggesting the potential of NDs toward the application of monitoring the brain cell behaviors in super-resolution.

## Supplementary Information

Additional file 1.

Additional file 2.

## Data Availability

All data generated or analyzed during this study are included in this published article and its supplementary information file.

## Abbreviations

CNS: Central nervous system
NA: Numerical aperture
PSF: Point spread function
STORM: Stochastic optical reconstruction microscopy
dSTORM: Direct stochastic optical reconstruction microscopy
CNP: Carbon nanoparticle
BBB: Blood–brain barrier
ND: Nanodiamond
NV: Nitrogen-vacancy
ROS: Reactive oxygen species
TPM: Two-photon microscopy
DI: Deionized
SAED: Selected-area electron diffraction
FFT: Fast Fourier transform
FOV: Field of view
DPBS: Dulbecco’s phosphate-buffered saline
PMT: Photomultiplier tube
GM: Growth medium
DM: Differentiation medium
DIV: Days in vitro
CCK-8: Cell counting Kit-8
LDH: Lactate dehydrogenase
DCFDA: Dichlorofluorescein diacetate
FITC: Fluorescein isothiocyanate
DAPI: 4,6-Diamidino-2-phenylindole
TRITC: Tetramethylrhodamine isothiocyanate
H&E: Hematoxylin and eosin
ESIPT: Excited-state intramolecular proton transfer
PEG: Polyethylene glycol
AIE: Aggregation-induced emission
NIR-II: Near-infrared II
