# Supplementary figures and images for: A systematic study on the use of multifunctional nanodiamonds for neuritogenesis and super-resolution imaging
Source: Biomater Res. 2023 Apr 27;27:37. doi: 10.1186/s40824-023-00384-9 (PMC10134586; doi:10.1186/s40824-023-00384-9)

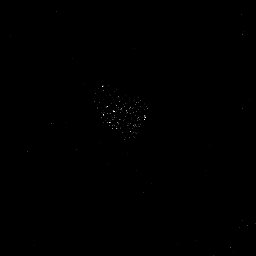

Supplement: Supplementary file 1 — Additional file 1. [file 40824_2023_384_MOESM1_ESM.gif]
