# Supplementary material for: A systematic study on the use of multifunctional nanodiamonds for neuritogenesis and super-resolution imaging
Source: Biomater Res. 2023 Apr 27;27:37. doi: 10.1186/s40824-023-00384-9 (PMC10134586; doi:10.1186/s40824-023-00384-9)
Supplement: Supplementary file 2 — Additional file 2. [file 40824_2023_384_MOESM2_ESM.docx]

***Supplementary data***

**A systematic study on the use of multifunctional nanodiamonds for neuritogenesis and super-resolution imaging**

**Jaeheung Kim^1†^, Moon Sung Kang^1†^, Seung Won Jun^2^, Hyo Jung Jo^1^, Dong-Wook Han^1,3^* and Chang-Seok Kim^1,4^***

**^1^**Department of Cogno-Mechatronics Engineering, Pusan National University, Busan 46241, Republic of Korea

**^2^**Agency for Defense Development, Ground Technology Research Institute, Daejeon 34186, Republic of Korea

^3^Bio-IT Fusion Technology Research Institute, Pusan National University, Busan 46241, Republic of Korea

**^4^**Engineering Research Center for Color-Modulated Extra-Sensory Perception Technology, Pusan National University, Busan 46241, Republic of Korea

* Correspondence: [nanohan@pusan.ac.kr](mailto:nanohan@pusan.ac.kr) (D.-W.H.); [ckim@pusan.ac.kr](mailto:ckim@pusan.ac.kr) (C.-S.K.)

^†^ These authors contributed equally to this work.


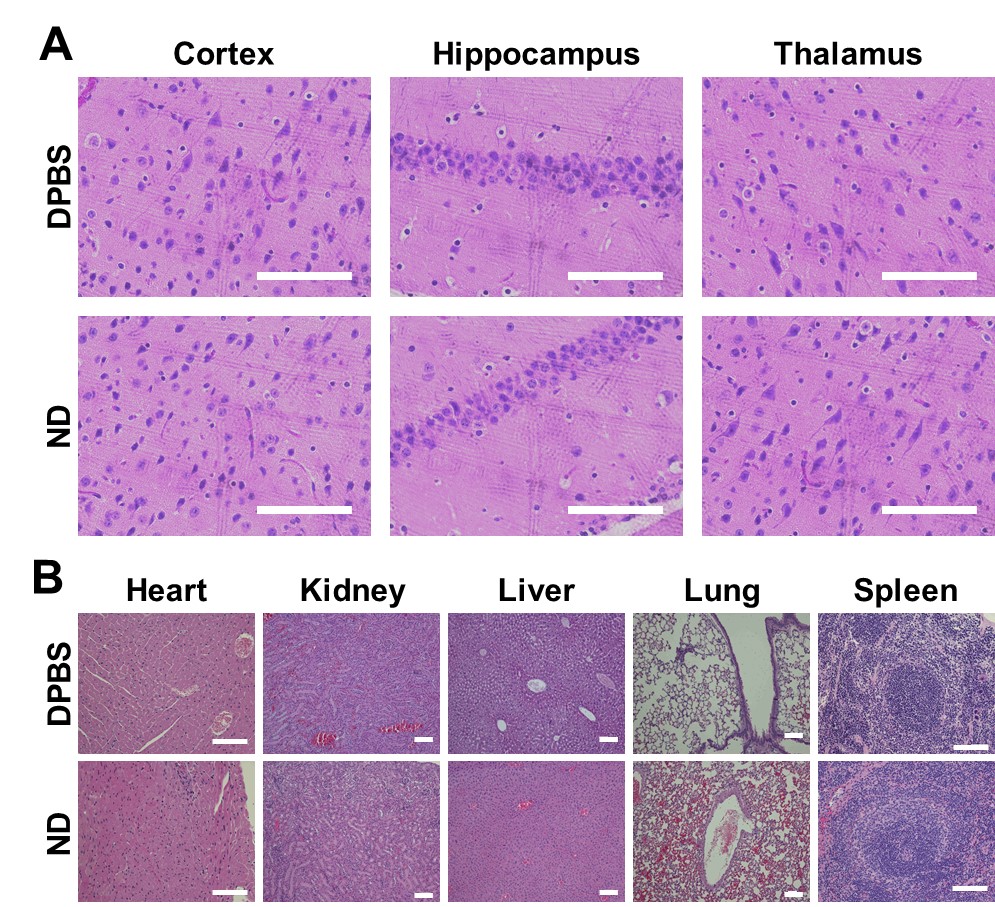


**Fig. S1.** DPBS or ND-injected H&E-stained organs of the mouse after 10 d post-injection. Microscopic examination of horizontal sections of H&E-stained (A) brains including cortex, hippocampus, and thalamus, and (B) main organs including heart, kidney, liver, lung, and spleen. Scale bars without value denote 100 µm.
